# 

**Published:** 1891-11-07

**Authors:** 


PRICE ONE PENNY.

o. 267, Vol. XI.—November 7th, 1891.

egistered at the General Post Office as a Newspaper for transmission in the United Kingdom and Abroad.]

WITH EXTRA NURSING SUPPLEMENT.

Per Annum, 6s. 6d.; post free

Monthly Parts, 6d.; post free 8d.

Ditto per Annum, 8s., post free.

### THE HOSPITAL

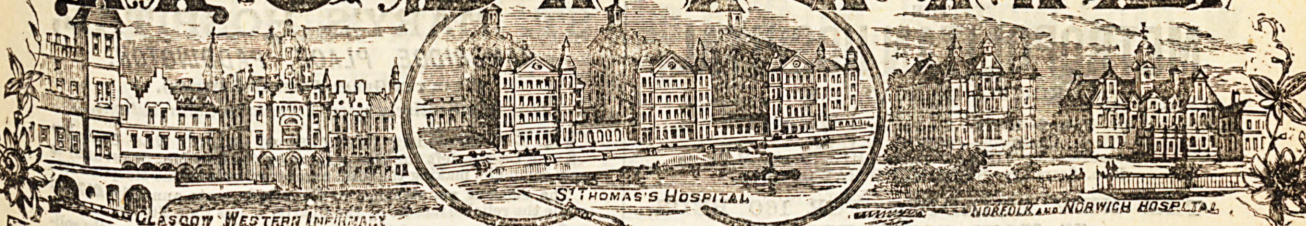

A WEEKLY JOURNAL OF
### Science, Medicine, Nursing and Philanthropy.

SATURDAY, NOVEMBER 7th, 1891.

**The Increase of Diabetes** - 61

**Passing Topics.**—"Tipping the Porter" - 62

**The Doctor's Armchair.**—The Cambridge Executioner - 63

**Medical History from the Earliest Times.**—By E. T. Withington, M.A., M. B. Oxon. I.—Introductory - 64

**Theosophy.**—Two Pleas for Mrs. Besant - 65

**Editor's Letter Box.**—A Puzzle - 65

**Notes and News** - 66

**Annotations.**—Unpractical London—Science, Religion, and Mariana in her Moated Grange—Surgeon Parke's Book - 68

**General Charities—Scraps and Gleanings** - 67

**The Practitioner's Mirror and Retrospect—**

MEDICINE —New Methods of Treatment in Phthisis, VI.—The Picot Method, 69; On Flushing - 70

SURGERY.—The Varieties of Lymphangitis - 71

**An Exhibition of Caterers** - 71

**The Student of Medicine.**—Syphilis: Its transmission and Treatment — Appointments — Vacancies — Notes from the Schools—Scholarships and Prizes—Football—Events for the Month of November

**EXTRA SUPPLEMENT—THE NURSING MIRROR.**

**En Passant.**—Short Items—Tombstones—American Gossip—Taunton Jubilee Institute—Glasgow Again—The Eastern Hospital - xxxi

**Lectures on Surgical Ward Work and Nursing.** By Alexander Miles, M.D. Edin., F.R.C.S.E., Lecture XXXVIII (*continued*) - xxxii

**Notes and Queries** - xxxii

**Examination Questions** - xxxiii

**For Reading to the Sick.**—Self-Control - xxxiii

**A Nurse in Natal** - xxxiv

**Christmas Competitions** - xxxiv

**Presentations** - xxxv

**Appointments** - xxxv

**Everybody's Opinion.**—Monthly Nurses—Male Nurses - xxxv

**Wants and Workers** - xxxv

**Off Duty.**—Bruce - xxxvi

**A Bed for a Sick Nurse** - xxxvi

**Amusements and Relaxation** - xxxvi

